# The First Attested Extraction of Ancient DNA in Legumes (Fabaceae)

**DOI:** 10.3389/fpls.2015.01006

**Published:** 2015-11-17

**Authors:** Aleksandar M. Mikić

**Affiliations:** Forage Crops Department, Institute of Field and Vegetable CropsNovi Sad, Serbia

**Keywords:** ancient DNA, bitter vetch, extraction, Fabaceae, legumes, pea

## Abstract

Ancient DNA (aDNA) is any DNA extracted from ancient specimens, important for diverse evolutionary researches. The major obstacles in aDNA studies are mutations, contamination and fragmentation. Its studies may be crucial for crop history if integrated with human aDNA research and historical linguistics, both general and relating to agriculture. Legumes (Fabaceae) are one of the richest end economically most important plant families, not only from Neolithic onwards, since they were used as food by Neanderthals and Paleolithic modern man. The idea of extracting and analyzing legume aDNA was considered beneficial for both basic science and applied research, with an emphasis on genetic resources and plant breeding. The first reported successful and attested extraction of the legume aDNA was done from the sample of charred seeds of pea (*Pisum sativum*) and bitter vetch (*Vicia ervilia*) from Hissar, southeast Serbia, dated to 1,350–1,000 Before Christ. A modified version of cetyltrimethylammonium bromide (CTAB) method and the commercial kit for DNA extraction QIAGEN DNAesy yielded several ng μl^-1^ of aDNA of both species and, after the whole genome amplification and with a fragment of nuclear ribosomal DNA gene 26S rDNA, resulted in the detection of the aDNA among the PCR products. A comparative analysis of four informative chloroplast DNA regions (*trnSG*, *trnK*, *matK*, and *rbcL*) among the modern wild and cultivated pea taxa demonstrated not only that the extracted aDNA was genuine, on the basis of mutation rate, but also that the ancient Hissar pea was most likely an early domesticated crop, related to the modern wild pea of a neighboring region. It is anticipated that this premier extraction of legume aDNA may provide taxonomists with the answers to diverse questions, such as leaf development in legumes, as well as with novel data on the single steps in domesticating legume crops worldwide.

## Ancient DNA

The term *ancient DNA* (aDNA) in its widest sense denotes any DNA extracted from ancient specimens either few centuries (Higuchi et al., 1984) or several millennia old (Pääbo, 1985). Since these pioneering breakthroughs in the field, an increasing number of aDNA enabled us comprehending biological and genetic changes over time, which provided us with direct insight into past genetic variation and clarified phylogenetic links between numerous extinct species (Stiller et al., 2006). In parallel with the progress of this novel discipline with a coined name paleogenetics, our knowledge on many unexpected and often completely misleading obstacles in properly interpreting the results of aDNA analysis has been accumulated. The major ones are mutations, caused by diverse post-mortem damages (Binladen et al., 2006), contamination, most often with modern DNA of the same or close species and responsible for frequently contrasting results of the analyses of the same material (Helgason et al., 2007), and fragmentation, being a consequence of the physical degradation of any DNA over time (Champlot et al., 2010).

## Plant aDNA

Reasonably enough, extracting aDNA from plants came somewhat later in comparison to those from humans and animals, especially mammals. One of such outstanding achievements brought to the daylight the DNA of an extinct plant from amber dated back to more than 20 million years ago (Poinar et al., 1993). The milestones such as this one represent an incredibly potent tool not only for exploring the plant evolution, but also for developing history of crop. This is most evident if the plant aDNA studies are integrated with the research on ancient human populations, archaeobotany of cultivated plants and historical linguistics assessing the origin and derivation of the ‘agricultural’ vocabulary. Such joint efforts may produce verily seminal discoveries (Mikić et al., 2014), such as casting much more light onto the language spoken by the first farmers in the world (Diamond and Bellwood, 2003), confirming that the bearers of the ‘agricultural revolution’ in Europe were immigrants from Near East (Haak et al., 2010) or making possible to concurrently follow the human migrations and language development (Balter and Gibbons, 2015) and assessing the connections among the well-established ethnolinguistic families at a significantly earlier time than conventionally considered (Gell-Mann et al., 2009; Mikić, 2012, 2015).

## Legumes

Legumes (Fabaceae Lindl.) are one of the richest existing plant families, comprising a large number of economically significant species used in human diets, animal feeding and non-food industries from time immemorial and with an extraordinary importance for the sustainability of natural resources stewardship (Rubiales and Mikić, 2015). Grain legumes or pulses, such as chickpea (*Cicer arietinum* L.), lentil (*Lens culinaris* Medik.), pea (*Pisum sativum* L.) or bitter vetch [*Vicia ervilia* (L.) Willd.] are not only among the first domesticated plant species (Tanno and Willcox, 2006), but also were used millennia before by both Neanderthals (Henry et al., 2011) and Paleolithic hunter-gatherers of then ice-bound Eurasia (Aura et al., 2005). By all these reasons, it may not be strange that the idea of a potential extraction and analysis of legume aDNA was conceived from the viewpoints of both basic science, such as the evolution of legumes, and applied research, with an emphasis on genetic resources and plant breeding (Medović et al., 2010).

## Legume aDNA

**FIGURE 1:**
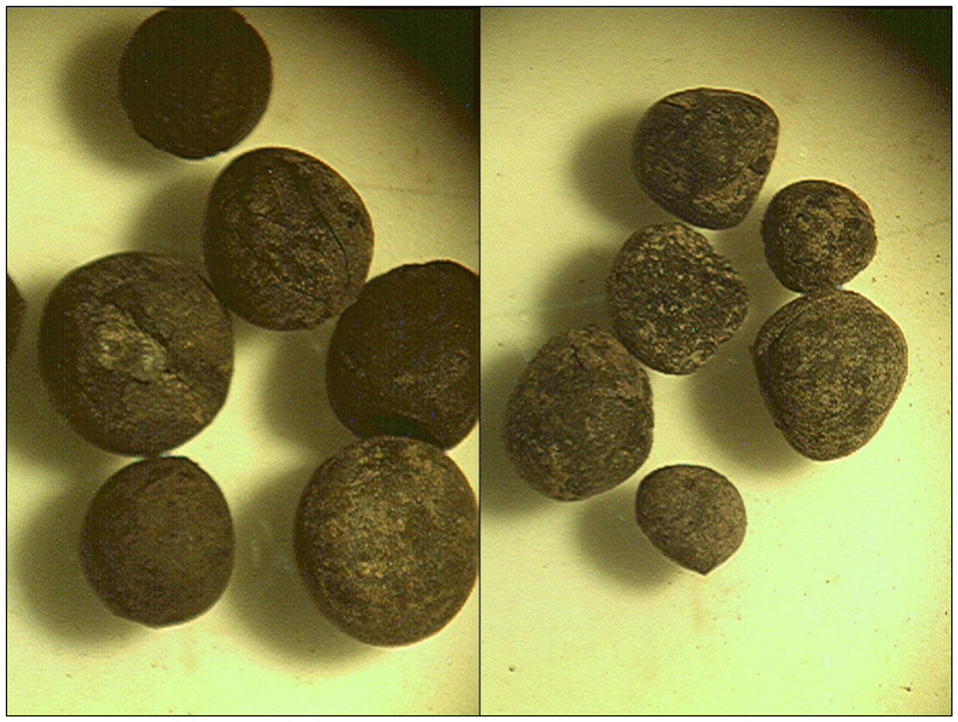
**Charred seeds of pea (left) and bitter vetch (right) from Hissar, south-east Serbia, cca 11th century BC**.

The first reported successful and attested extraction of the aDNA from some legume species was done from the charred seeds of pea and bitter vetch from a fortified hill fort Hissar, near the modern town of Leskovac in southeast Serbia (Medović et al., 2011). Hissar belonged to the Early Iron Age cultural group of Brnjica and most likely was the northernmost point of then Hellenic-based civilisation, dated back to between 1,350 BC and 1,000 BC. What makes this archeological site unique is a rather unusual structure of its archaeobotanical finds. Unlike a vast majority of settlements from the earliest Neolithic onwards, where the material evidence of cereals is regularly much more numerous the one of grain legumes, the excavations at Hissar brought to the daylight nearly 2,600 charred pea and more than 3,000 charred bitter vetch seeds (**Figure 1**), with only few dozens of the charred seeds of cereals, other crops and weeds (Medović et al., 2011).

**FIGURE 2:**
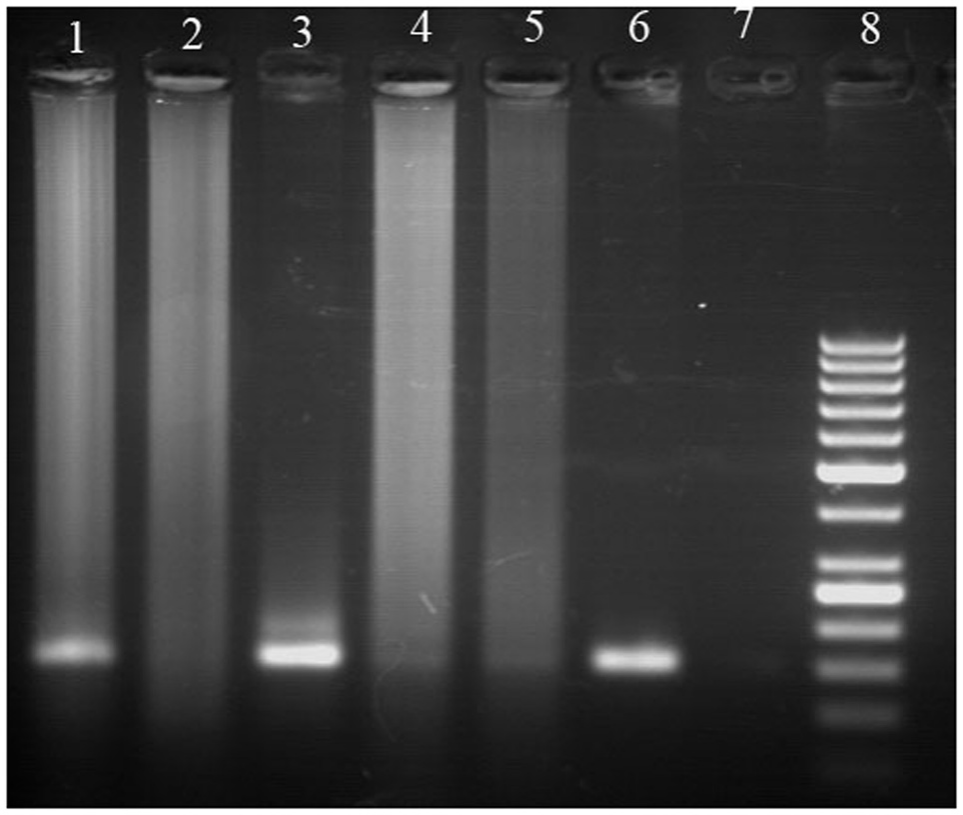
**Polymerase chain reaction (PCR) products obtained after amplification of ancient and modern DNA from pea and bitter vetch with 26S rDNA primers (Jovanović et al., 2011): (1) ancient pea DNA extracted using commercial kit, (2) ancient pea DNA extracted using cetyltrimethylammonium bromide (CTAB) method, (3) modern pea DNA, (4) ancient bitter vetch DNA extracted using commercial kit, (5) ancient bitter vetch DNA extracted using CTAB method, (6) modern bitter vetch DNA, (7) negative control, (8) DNA size marker (50 bp Fermentas)**.

The aDNA extraction from the Hissar charred pea and bitter vetch seeds was done by two different methods, namely the modified version of the cetyltrimethylammonium bromide (CTAB) method and the commercial kit for DNA extraction QIAGEN DNAesy. The modification of the CTAB method included increasing the concentration to 3%, in order to improve the osmolarity and obtain better seed disruption, as well as using an insoluble polyvinylpolypyrrolidone (PVPP), remarkably efficient in absorbing polyphenols during purifying DNA and thus preventing them from deactivating proteins and inhibiting the downstream reactions like polymerase chain reaction (PCR). Since the yield of the extracted aDNA from the charred legume seeds was of a magnitude of several ng μl^-1^, the whole genome amplification was applied, together with a fragment of nuclear ribosomal DNA gene, 26S rDNA, eventually resulting to the detection of the aDNA among the PCR products (**Figure 2**). This was an evidence that the aDNA from charred grain legume seeds, such as those from Hissar, may be relatively easily extracted using a commercial kit, as well as that the DNA fragments, such as 26S rDNA, can be amplified by PCR and found useful for further archaeobotanical and paleogenetic analyses (Jovanović et al., 2011).

At the same time this premiere analysis of a legume aDNA was done, a rediscovery of wild relatives of pea, especially so-called ‘tall’ pea [*P. sativum* subsp. *elatius* (Steven ex M. Bieb.) Asch. & Graebn.] was reported (Zlatković et al., 2010). A population of this pea taxon was located in a wood on the slopes of the Mount Kozjak, quite close to the monastery of Saint Prohor of Pčinja from 10th century, and some 150 km south from Hissar. A collecting expedition to the targeted region in May 2011 resulted in obtaining plant material from the modern ‘tall’ pea population and several other pea wild relatives, such as *P. abyssinicum* A. Braun and *P. fulvum* Sm., and their comparison to the ancient pea from Hissar. For this purpose, four specific phylogenetically informative fragments in all the pea taxa were used, selected on the basis of previous analysis of wild and cultivated species of pea and other genera of the tribe *Vicieae*, namely chloroplast DNA loci *trnS(GCU)-trnG(UCC)* intergenic spacer, *tRNA-Lys (trnK)* partial sequence, maturase K *(matK)* and ribulose-1,5-bisphosphate carboxylase/oxygenase large subunit *(rbcL)* partial gene regions (Smýkal et al., 2014a). The charred seeds were carefully chosen for the analysis, avoiding any possibility of contamination with the DNA of modern pea or related genera, and their DNA was extracted from bulk, due to their age, a considerable degree of physical damages and assumingly low amount of the preserved DNA. On the basis of the well-documented deamination of cytosine (and 5-methyl cytosine) to uracil (and thymine), associated with *post-mortem* damage, and since the majority of the substitutions were of the type 2 transitions, it was evident that the DNA extracted from the charred pea seeds from Hissar was a genuine aDNA. Both phenotypic and molecular data suggest that the Hissar pea most likely was an early domesticated pea, with a possibility that it was first collected by the town inhabitants or their unknown predecessors in the surrounding wild flora and then gradually cultivated. Still at a level of speculation, the Hissar pea could have colored flower and pigmented seed coat and probably adapted to germinating in the autumn and being winter hardy (Smýkal et al., 2014b).

## Perspectives

The premier known attested extraction of the ancient DNA from the charred grain legume seeds opens numerous possibilities for further research in the most diverse directions. Since the botanical importance of the legume plant family, some future aDNA analyses may provide the global taxonomic community with at least few answers to certain still unsolved questions, such as the leaf development in legumes, in other words, if the compound leaf present in the majority of legume species evolved from the simple one or *vice versa* and the origin of tendrils. It may also give a clearer insight into the whole family evolution and mutual relationship among diverse taxa. In addition, the economic significance of legumes may be enriched with novel data on the single steps in domesticating each legume crop and their links with wild relatives. Yet, there remains the fact how, despite sundering hundreds of miles and hundreds of decades, the ancient Hissar pea and the modern Kozjak pea are related to each other, almost surrealistically untouched by space and time and witnessing how much is left for us to seek, find and comprehend.

## Author Contributions

AM conceived the idea of extracting legume aDNA and wrote the manuscript.

## Conflict of Interest Statement

The authors declare that the research was conducted in the absence of any commercial or financial relationships that could be construed as a potential conflict of interest.
